# Altered Empathy Processing in Frontotemporal Dementia

**DOI:** 10.1001/jamanetworkopen.2024.48601

**Published:** 2024-12-03

**Authors:** Olof Lindberg, Tie-Qiang Li, Cecilia Lind, Susanna Vestberg, Ove Almkvist, Mikael Stiernstedt, Anita Ericson, Nenad Bogdanovic, Oskar Hansson, Luke Harper, Eric Westman, Caroline Graff, Theofanis Tsevis, Peter Mannfolk, Håkan Fischer, Gustav Nilsonne, Predrag Petrovic, Lars Nyberg, Lars-Olof Wahlund, Alexander F. Santillo

**Affiliations:** 1 Division of Clinical Geriatrics, Centre for Alzheimer Research, Department of Neurobiology, Care Sciences and Society, Karolinska Institute, Stockholm, Sweden; 2 Department of Clinical Science, Intervention, and Technology, Karolinska Institute, Stockholm, Sweden; 3 Department of Medical Radiation and Nuclear Medicine, Karolinska University Hospital, Stockholm, Sweden; 4 Department of Community Medicine and Rehabilitation—Geriatrics, Umeå University, Umeå University, Umeå, Sweden; 5 Department of Psychology, Lund University, Lund, Sweden; 6 Department of Psychology, Stockholm, Sweden; 7 Umeå Center for Functional Brain Imaging (UFBI), Umeå University, Umeå, Sweden; 8 Clinical Memory Research Unit, Department of Clinical Sciences Malmö, Lund University, Lund, Sweden; 9 Memory Clinic, Skåne University Hospital, Lund, Sweden; 10 Karolinska University Hospital, Stockholm, Sweden; 11 Department of Neurobiology, Care Sciences and Society, Division of Neurogeriatrics, Center for Alzheimer Research, Karolinska Institutet, Solna, Sweden; 12 Department of Medical Imaging and Physiology, Skåne University Hospital, Lund, Sweden; 13 Stockholm University Brain Imaging Centre (SUBIC), Stockholm, Sweden; 14 Aging Research Center, Karolinska Institutet, Stockholm, Sweden; 15 Division of Psychology, Department of Clinical Neuroscience, Karolinska Institutet, Stockholm, Sweden; 16 Center for Psychiatric Research, Department of Clinical Neuroscience, Karolinska Institute, Stockholm, Sweden; 17 Center for Cognitive and Computational Psychiatry, Department of Clinical Neuroscience, Karolinska Institute, Stockholm, Sweden; 18 Department of Diagnostics and Intervention, Umeå University, Umeå, Sweden; 19 Department of Medical and Translational Biology, Umeå University, Umeå, Sweden

## Abstract

This case-control study examines differences in empathetic response of patients experiencing behavioral variant frontotemporal dementia using functional magnetic resonance imaging.

## Introduction

Loss of empathy is a core symptom of behavioral variant frontotemporal dementia (bvFTD).^1^ In particular, the affective aspect of empathy appears to be independent of decrease in the other socioemotional abilities and general cognition in bvFTD.^2^ We used an established functional magnetic resonance imaging (MRI) paradigm^3^ to assess bvFTD-related alterations in brain responses during empathy for pain (EFP) in a case-control study.

## Methods

We studied 28 persons with bvFTD and 28 cognitively normal controls (eFigure 1 in Supplement 1). The study was approved by the local ethics review board in Stockholm. Individuals were recruited from 2015 to 2022. BvFTD was diagnosed according to established international criteria.^1^ The Interpersonal Reactivity Index (IRI)^4^ was used to measure empathic function. We followed the Strengthening the Reporting of Observational Studies in Epidemiology (STROBE) reporting guidelines. Written informed consent was obtained from all participants.

Acquisition parameters are described in eMethods in Supplement 1. Analysis of task-based functional MRI (fMRI) data was conducted with FEAT version 6.00 (FSL, FMRIB). The fMRI paradigm is displayed in the Figure, A and B.

**Figure.**
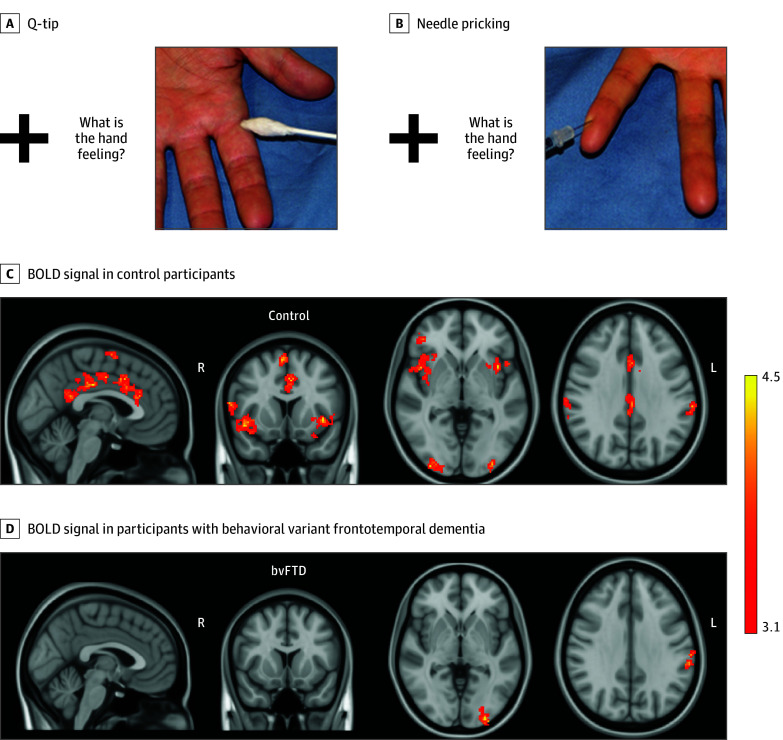
Experimental fMRI Paradigm and BOLD Signal Change in the Empathy of Pain Contrast The figure shows 1 example of the 20 images of Q-tip touching a hand (A) and 1 example of the 20 images of a needle pricking a hand (B) displayed during the functional magnetic resonance imaging (fMRI) experiment. A fixation cross is displayed for 3 to 5 seconds, followed by the text “What is the hand feeling?” displayed for 3 seconds. Subsequently the image is displayed for 3.5 seconds, after which a black image is displayed for 4.5 seconds before a new cycle is initiated with a new fixation cross. Areas with significant increased blood oxygen level–dependent (BOLD) signal in control participants (C) and in patients with behavioral variant frontotemporal dementia (D) in the empathy of pain contrast. The color bar displays *z*-scores (red *z* > 3.1, yellow *z* = 4.5). From left to right sagittal, coronal, and axial slices at MNI coordinates *x* = −2, *y* = 12, *z* = −2. Last axial slice at MNI coordinates *x* = 62, *y* = −32, *z* = 32.

Student *t* tests were used to test differences between patients and controls on demographic variables that were normally distributed, otherwise Mann-Whitney *U* tests were used. A *P* value less than .05 was considered significant.

Group-level fMRI statistics were evaluated using FMRIB Local Analysis of Mixed Effects (FLAME 1&2). A statistical threshold was set to *z* > 2.3 at individual level and *z* > 3.1 with *P* < .05 at group-level using whole-brain cluster–wise correction. Brain responses related to EFP were analyzed by subtracting blood oxygen level–dependent (BOLD) signal in the control condition from signal in the pain condition (Figure, A and B).

We used 2 regions-of-interest (ROI) approaches, one based on a meta-analysis on areas commonly activated during EFP^5^ and one based on the control’s activation pattern (CA-ROI) during EFP (eFigures 2 and 3 in Supplement 1). The CA-ROI was used to study associations with IRI subscales as it reflected the expected normal activation pattern elicited by the empathy task (eFigure 3 in Supplement 1). Detailed description of methods is reported in eMethods in Supplement 1.

## Results

There were no differences between patients and controls regarding age or years of education (mean [SD] age: 66.7 [6.7] years vs 67.6 [7.4] years) (Table). Significantly increased BOLD signal during EFP was observed in 12 areas in controls (Figure, C) and in 2 areas in bvFTD (Figure, D). Patients displayed reduced BOLD signal under the affective empathy ROI (mean change during EFP: control, 20.86%; 95% CI, 10.52% to 31.20%; bvFTD, −1.26%; 95% CI, −11.60% to 9.07%; *P* = .004), but not under the cognitive empathy ROI. BOLD signal in the CA-ROI during EFP was significantly positively correlated with the control participants’ self-rating of their empathic concern in the IRI (*r* = 0.61; 95% CI, 0.21-0.83), and with informants’ ratings of patients’ empathic concern (*r* = 0.50; 95% CI, 0.06-0.78; *P* = .03).

**Table.** Demographic, Interpersonal Reactivity Index, and Neuropsychological Data

Characteristics	Participants, mean (SD)	
	Control (n = 28)	bvFTD (n = 28)
Gender, No. (%)		
Men	12 (43)	13 (46)
Women	16 (57)	15 (54)
Site, No. (%)		
Lund	13 (46)	21 (75)
Karolinska	15 (54)	2 (7)
Umeå	0	5 (18)
Age, y	67.6 (7.4)	66.7 (6.7)
Education, y	13 (3.1)	12 (2.97)
MMSE score	29.7 (0.58)	24.71 (4.97)^a^
IRI-ratings		
Fantasy self-rating	2.99 (0.71)	2.74 (0.82)
Empathic concern self-rating	3.11 (0.28)	3.28 (0.75)
Perspective taking self-rating	3.50 (0.37)	3.44 (0.71)
Personal distress self-rating	3.05 (0.42)	3.03 (0.97)
Fantasy informants’ rating	NA	2.47 (0.56)
Empathic concern informants’ rating	NA	3.14 (0.35)
Perspective taking informants’ rating	NA	2.75 (0.36)
Personal distress informants’ rating	NA	2.90 (0.51)
Neuropsychological tests, *z* score		
Digit span forward	0 (1)	−0.41 (0.87)
Digit-span backward	0 (1)	−0.58 (1.01)
Trail making A	0 (1)	−2.63 (4.06)^b^

Abbreviations: bvFTD, behavioral variant frontotemporal dementia; IRI, Interpersonal Reactivity Index; MMSE, Mini Mental State Examination; NA, not applicable.

^a^ Mann-Whitney U test, *P* < .01.

^b^ Controls performed significantly better than patients with bvFTD, *P* for between-group difference = .002.

## Discussion

We found that in a task-based fMRI empathy for pain paradigm, patients with bvFTD exhibit reduced brain response in regions known to be of central importance for empathy processing in the healthy human brain and affected early by the diverse neuropathological processes in the bvFTD syndrome. Importantly, the magnitude of empathy-related neural activity was correlated with the patients’ ability to experience empathy, as judged by the individuals living with the patients affected by bvFTD. Limitations of this study are the use of multiple MRI scanners, the inclusion of patients with both sporadic and genetic bvFTD, and the lack of neuropathological verification of the bvFTD diagnoses, that are addressed in sensitivity analyses.

## Notes

Supplement 1.
eMethods.
**eFigure 1.** Flowchart Displaying Inclusion Procedures of Patients and Controls **eFigure 2.** Spheres Located at Coordinates With Peak Activation in Affective and Cognitive Empathy in the Meta-Analysis by Fan et al **eFigure 3.** The 12 Areas Included in the Control Activation ROI
eReferences

Supplement 2.
Data Sharing Statement
