# Supplementary material for: Altered Empathy Processing in Frontotemporal Dementia
Source: JAMA Netw Open. 2024 Dec 3;7(12):e2448601. doi: 10.1001/jamanetworkopen.2024.48601 (PMC11615710; doi:10.1001/jamanetworkopen.2024.48601)
Supplement: Supplement 1. — eMethods. eFigure 1. Flowchart Displaying Inclusion Procedures of Patients and Controls eFigure 2. Spheres Located at Coordinates With Peak Activation in Affective and Cognitive Empathy in the Meta-Analysis by Fan et al eFigure 3. The 12 Areas Included in the Control Activation ROI eReferences [file jamanetwopen-e2448601-s001.pdf]

Supplemental Online Content

Lindberg O, Li T, Lind C, et al. Altered empathy processing in frontotemporal dementia. *JAMA Netw Open.* 2024;7(12):e2448601. doi:10.1001/jamanetworkopen.2024.48601

eMethods

eFigure 1. Flowchart displaying inclusion procedures of patients and controls

eFigure 2. Spheres Located at Coordinates With Peak Activation in Affective and Cognitive Empathy in the Meta-Analysis by Fan et al

eFigure 3. The 12 Areas Included in the Control Activation ROI

eReferences

This supplemental material has been provided by the authors to give readers additional information about their work.

© 2024 Lindberg O et al. *JAMA Network Open.*

eMethods

The interpersonal reactivity index

The interpersonal reactivity index (IRI) is a 28-items questionary that aims to measure a person´s ability to react to the observed experience of another person with four subscales: One) Perspective taking (PT), the tendency to spontaneously adopt the psychological point of view of others, two) Fantasy (FS), the respondent´s tendencies to transpose themselves imaginatively into the feelings and actions of fictitious characters in books, movies or plays, three) Empathic concern, the "other-oriented" feelings of personal unease and anxiety for others in distress, and four) Personal Distress (PD), that taps in on feelings of personal anxiety and unease in tense interpersonal settings.[1] Rating is performed along a Likert scale with five levels. One characteristic of patients with bvFTD is loss of insight.[2] Therefore, in addition to patients rating themselves on the IRI an informant (in most cases a close relative living with the patient or someone with regular and frequent interaction with the patient) rated the patients, as customary in research on bvFTD using the IRI. [3-5] Controls rated themselves. Pearson´s correlation was used to study the relationship between ratings on the IRI subscale and mean BOLD signal during EFP.

Sites in Sweden from which participants were recruited.

1) Skåne University Hospital in Lund.
   Entrégatan 7
   222 42 Lund

2) Norrlands University Hospital of Umeå.
   Daniel Naezéns väg
   907 37 Umeå

© 2024 Lindberg O et al. *JAMA Network Open.*

3) Karolinska University Hospital.
Hälsovägen 13
141 57 Huddinge

Additional inclusion criteria for controls and patients with bvFTD

Inclusion criteria were a diagnosis of bvFTD according to International Behavioral Variant FTD Consortium Criteria[2] (either possible, probable or definite bvFTD) following multidisciplinary assessment including clinical examination, caregiver interview, clinical neuropsychological examination, neuroimaging and lumbar puncture. Cerebrospinal fluid (CSF) was analyzed for amyloid β, tau and phosphorylated tau (p-tau). Due to the cognitive demands of the task-based paradigm only individuals with mild

dementia as specified by a clinical dementia rating (CDR) ≤ 1,[6] or a mini mental status examination (MMSE) ≥ 21 were included. [7] Additional inclusion criteria included the availability of a reliable informant, and proficiency in the Swedish language was required of both the patients and informants. Participants with a combination of CSF amyloid β (either amyloid β 42 or amyloid β 42/40 ratio) below and CSF p-tau above local laboratory reference values, indicative of Alzheimer´s disease, were excluded from study. In addition to neuropsychological testing performed during clinical assessment of included patients, did a majority patients and controls perform a set of tests as a part of the present study (Table 1 & Supplement 1 eFlowshart). Global cognition was measured using the MMSE,[7] executive function/working memory was assessed with the Digit Span backward tests, attention was assessed with the Digit Span forward [both from WAIS-IV, [8]] and psychomotor speed was assessed using the Trail Making Test part A.[9]

A convenience sampled control group of healthy individuals were included.

© 2024 Lindberg O et al. *JAMA Network Open.*

The Swedish text that are displayed during the fMRI paradigm

The text “What is the hand feeling” is in the Swedish version used in this paradigm translated into “Vad känner handen?”.

Image acquisition

Structural and functional MRI data was acquired with a 64-channel head coil in two 3T Prisma MRI scanners (Siemens MAGNETOM Prisma, Siemens Healthcare, Erlangen, Germany) in Lund and in Stockholm, and one Discovery MR750 (General Electric system, GE Healthcare, United States) using a 32-channel head coil in Umeå.

Structural MRI

In the Prisma MRI scanners structural 3-D T1-weighted images were acquired with a voxel size= 1x1x1.2 mm^3, with an inversion time (TI)=900 milli seconds (ms), repetition time (TR)=7100 ms, echo time (TE)=2.98 ms and a flip angle (FA) of 9 degrees. In the GE-MRI scanner the T1was acquired with a SPGR 3-D sequence with a TR=8156 ms TE =3.18, TI=450 ms, a FA 12 degrees, and a voxel dimension of 1x1x1mm^3.

Task-based fMRI

In the Prisma scanners task-based fMRI images were acquired with a TR=3000 ms TE=34 ms, FA 90 degrees, with an in-plane resolution of 2.3mm*2.3mm and slice thickness of 2.3 mm. In the GE MRI scanner task-based fMRI images were acquired with a TR=3000 ms TE=34 ms, FA=85 degrees, with an in-plane resolution of 2.38*2.38 and slice thickness of 2.3 mm.

© 2024 Lindberg O et al. *JAMA Network Open.*

Image quality control

Quality control was carried out on all MRI data according to previous described procedures, [8] and data management and processing were done through our in-house database system. [9] During the experimental fMRI acquisition, all patients and controls had a relative displacement of less than a half voxel (>1.15 mm) and an absolute displacement of less than a voxel (>2.33 mm). The mean relative movements (between one acquired volume to another) in controls was 0.08 mm and in patients 0.11 mm. This difference was not significant (p = 0.07).

Structural MRI analysis in FreeSurfer

Cortical reconstruction and volumetric segmentation of subcortical volumes were performed on T1 3D images using Freesurfer 6.0.0 image analysis pipeline, which is documented and freely available for download online (http://surfer.nmr.mgh.harvard.edu/). The technical details of these procedures are described in prior publications, which are listed at https://surfer.nmr.mgh.harvard.edu/fswiki/FreeSurferMethodsCitation. Briefly, the whole-brain T1-weighted images underwent a correction for intensity homogeneity, skull striping, and segmentation into GM and white matter (WM). Cortical thickness was measured as the distance from the gray/white matter boundary to the corresponding pial surface. Subcortical segmentation and assessment of intracranial volume was also performed in Freesurfer. Reconstructed data sets were visually inspected for accuracy, and segmentation errors were corrected. The FreeSurfer analysis was performed to evaluate whether decreased cortical thickness was associated with decreased BOLD signal during EFP.

© 2024 Lindberg O et al. *JAMA Network Open.*

Analysis of task-based functional MRI

Analysis of task-based fMRI data was performed using FEAT v6.00 (FSL, FMRIB, Oxford, UK). Raw fMRI scans were pre-processed before statistical analysis using the following steps: motion correction using MCFLIRT, [10] removal of nonbrain tissue using BET,[11] spatial smoothing using a full width at half maximum of 5mm gaussian kernel. Time series statistical analysis and improved linear model (FILM) with local autocorrection was also performed. [12] Finally, images were registered to the structural image and then to standard MNI space using FLIRT affine registration with 12 degrees of freedom. Relative displacement (head motion between consequentially acquired volumes) and absolute displacement (head motion in relation to the reference volume) was reported in FEAT.

Whole brain fMRI analysis

Group-level fMRI analysis was performed using FMRIB Local Analysis of Mixed Effects [FLAME 1&2, [13,14]]. Group level z (gaussianized t) statistical images were performed using a threshold of $z>3.1$, $p<0.05$ (whole-brain cluster-wise-corrected data). At individual level, z (gaussianized t) statistical images were produced. Values of $z>2.3$, $p<0.05$ (whole-brain cluster-wise-corrected data) was considered significant. The EFP contrast (pain condition minus control condition) was evaluated using the ROI approach. At whole brain level no significant differences were found between groups.

The meta-analysis ROIs

We used a regions-of-interest (ROI) approach, in which two ROIs were defined on basis of

© 2024 Lindberg O et al. *JAMA Network Open.*

findings in a metanalysis.[15] EFP was in this meta-analysis divided into an affective domain (affective empathy defined as the vicarious experience of other's sensorimotor or emotional state) and a cognitive domain (cognitive empathy, largely synonymous with "theory of mind" or "mentalizing", defined as the ability to understand the perspective of another person while maintaining a distinction between one´s own and the other person´s mental state).[15] Mean BOLD signal was extracted under areas that were found to have significantly BOLD signal during affective empathy (red spheres eFigure 1) and cognitive empathy (yellow spheres) in this meta-analysis (efigure 1).

© 2024 Lindberg O et al. *JAMA Network Open.*

eFigure 1. Flowchart displaying inclusion procedures of patients and controls

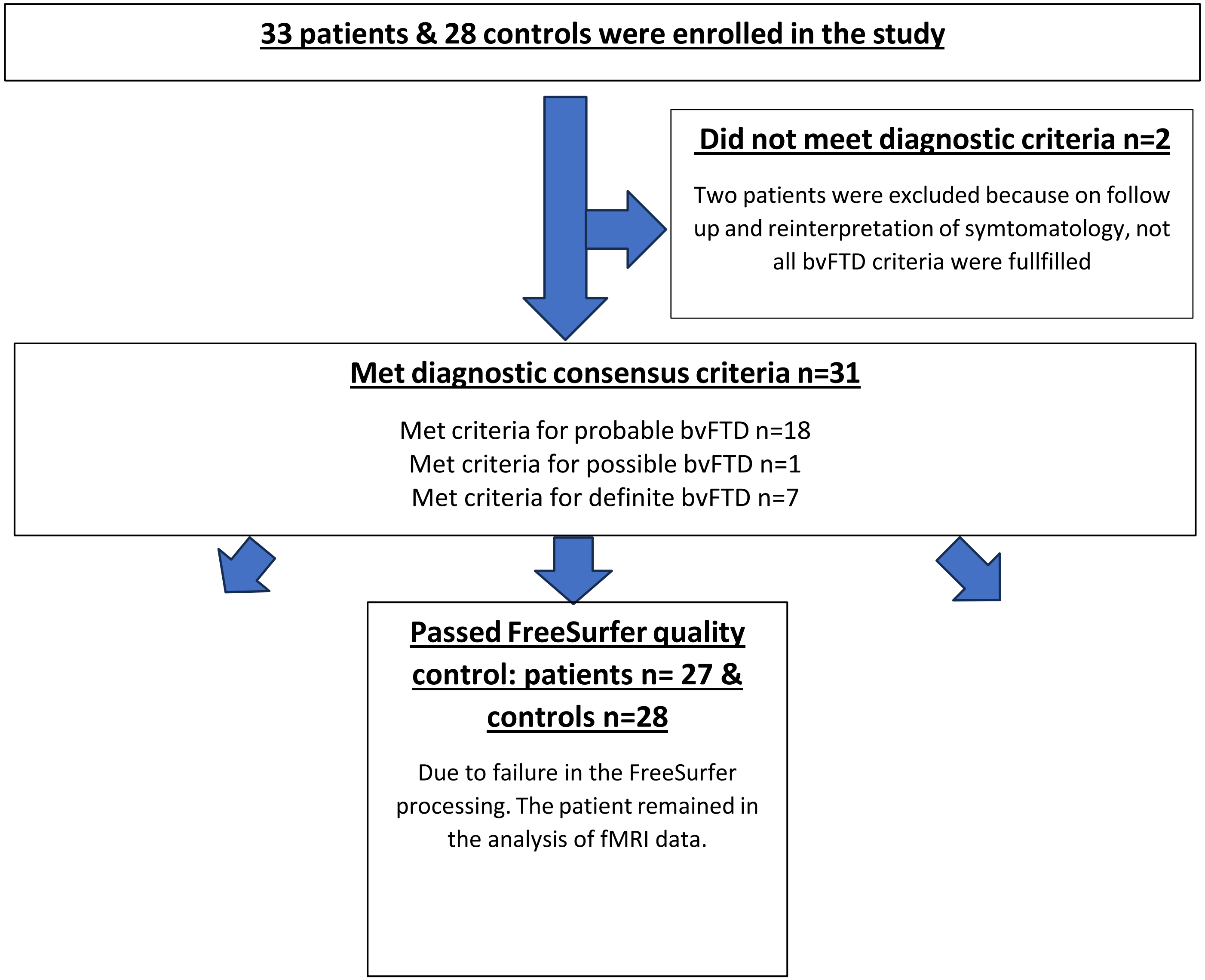

© 2024 Lindberg O et al. *JAMA Network Open.*

Passed fMRI quality control: patients n=28 & controls n=28

fMRI data was excluded for the following reasons:

1) Had percent signal change more than 4 standard deviation (SD) above patient with nearest observation (& 6SD above mean).
2) Technical problems connecting the head coil during scanning.
3) Upgrade of scanner (from Trio to Prisma).

Number that performed neuropsychology tests

MMSE: 28 BvFTD & 28 controls
Digitspan forward: 17 bvFTD & 20 controls
Digitspan backward: 20 bvFTD & 20 controls
Trail-making A: 19 bvFTD & 20 controls
IRI self-rating: bvFTD 18 & 19 controls
IRI informants-rating: 19 bvFTD
Pain sensation: 18 bvFTD

© 2024 Lindberg O et al. *JAMA Network Open.*

eFlowchart: Patients with the behavioral variant of frontotemporal dementia (bvFTD, n=28) and healthy controls (n=28) were included in the study (Table 1). All definite bvFTD cases were either carriers of pathogenic mutations in the chromosome 9 open reading frame 72 (*C9orf72*) gene, or the microtubule-associated protein tau (*MAPT*) gene, in the progranulin (*GRN*) gene. According to consensus criteria, [2] 7 patients fulfilled criteria for definite bvFTD, 18 for probable and one for possible bvFTD. Two patients declined to undergo CSF analysis. Not all bvFTD did manage to perform the IRI. Patients performed worse than controls on the TrailMaking A test (Mann-Whitney U-test, U=92, p<0.01, controls n=21, bvFTD n=25, Z = -3.75) and on MMSE (U=22, p<0.01, controls n=16, bvFTD n=28, Z = -4.92; Table 1).

© 2024 Lindberg O et al. *JAMA Network Open.*

eFigure 2. Spheres located at coordinates with peak activation in affective & cognitive empathy in the meta-analysis by Fan et al.

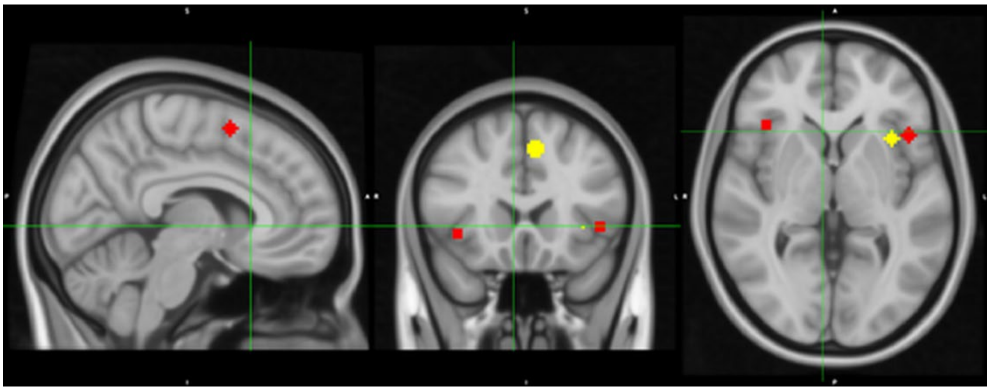

Red spheres (the Affective empathy ROI) located at peak activation specifically during affective empathy and yellow spheres (the cognitive empathy ROI) located at peak activation specifically during cognitive empathy (Table 2 in [15]).

© 2024 Lindberg O et al. *JAMA Network Open.*

eFigure 3. The 12 areas included in the control activation ROI

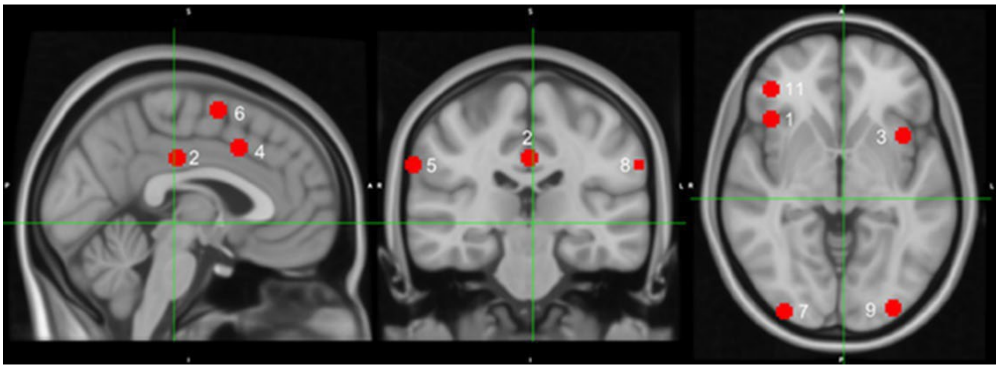

The figure displays 12 task-defined 5mm spherical ROIs located at peak activation in the 12 areas with significant activation in empathy of pain in controls. Mean BOLD-signal was extracted for the 12 ROIs (the CA-ROI). The sphere located in the right frontal pole (number 12), is not visible at image above.

© 2024 Lindberg O et al. *JAMA Network Open.*

eReferences

1. Davis MH. *A Multidimensional Approach to Individual Differences in Empathy*. vol 10. JSAS, Catalog of Selected Documents in Psychology. 1980.
2. Rascovsky K, Hodges JR, Knopman D, et al. Sensitivity of revised diagnostic criteria for the behavioural variant of frontotemporal dementia. *Brain : a journal of neurology*. Sep 2011;134(Pt 9):2456-77. doi:10.1093/brain/awr179
3. Dermody N, Wong S, Ahmed R, Piguet O, Hodges JR, Irish M. Uncovering the Neural Bases of Cognitive and Affective Empathy Deficits in Alzheimer's Disease and the Behavioral-Variant of Frontotemporal Dementia. *Journal of Alzheimer's disease : JAD*. May 30 2016;53(3):801-16. doi:10.3233/JAD-160175
4. Rankin KP, Gorno-Tempini ML, Allison SC, et al. Structural anatomy of empathy in neurodegenerative disease. *Brain : a journal of neurology*. Nov 2006;129(Pt 11):2945-56. doi:10.1093/brain/awl254
5. Rankin KP, Kramer JH, Miller BL. Patterns of cognitive and emotional empathy in frontotemporal lobar degeneration. *Cogn Behav Neurol*. Mar 2005;18(1):28-36.
6. Hughes CP, Berg L, Danziger WL, Coben LA, Martin RL. A new clinical scale for the staging of dementia. *The British journal of psychiatry : the journal of mental science*. Jun 1982;140:566-72.
7. Folstein M. "Mini-mental state". A practical method for grading the cognitive state of patients for the clinician. *Journal of psychiatric research*. 1975:189-198.
8. Wechsler D. *Wechsler Adult Intelligence Scale 4th edn*. Pearson, Inc (Harcourt Assessment, Swedish version); 2010.
9. Tombaugh TN. Trail Making Test A and B: normative data stratified by age and education. *Arch Clin Neuropsychol*. Mar 2004;19(2):203-14. doi:10.1016/S0887-6177(03)00039-8
10. Jenkinson M, Smith S. A global optimisation method for robust affine registration of brain images. *Med Image Anal*. Jun 2001;5(2):143-56. doi:10.1016/s1361-8415(01)00036-6
11. Smith SM. Fast robust automated brain extraction. *Human brain mapping*. Nov 2002;17(3):143-55. doi:10.1002/hbm.10062
12. Woolrich MW, Ripley BD, Brady M, Smith SM. Temporal autocorrelation in univariate linear modeling of FMRI data. *NeuroImage*. Dec 2001;14(6):1370-86. doi:10.1006/nimg.2001.0931
13. Beckmann CF, Jenkinson M, Smith SM. General multilevel linear modeling for group analysis in FMRI. *NeuroImage*. Oct 2003;20(2):1052-63. doi:10.1016/S1053-8119(03)00435-X
14. Woolrich MW, Jbabdi S, Patenaude B, et al. Bayesian analysis of neuroimaging data in FSL. *NeuroImage*. Mar 2009;45(1 Suppl):S173-86. doi:10.1016/j.neuroimage.2008.10.055
15. Fan Y, Duncan NW, de Greck M, Northoff G. Is there a core neural network in empathy? An fMRI based quantitative meta-analysis. *Neurosci Biobehav Rev*. Jan 2011;35(3):903-11. doi:10.1016/j.neubiorev.2010.10.009

© 2024 Lindberg O et al. *JAMA Network Open.*
