# Supplementary material for: Altered Empathy Processing in Frontotemporal Dementia
Source: JAMA Netw Open. 2024 Dec 3;7(12):e2448601. doi: 10.1001/jamanetworkopen.2024.48601 (PMC11615710; doi:10.1001/jamanetworkopen.2024.48601)
Supplement: Supplement 2. — Data Sharing Statement [file jamanetwopen-e2448601-s002.pdf]

Data Sharing Statement

Lindberg. Altered Empathy Processing in Frontotemporal Dementia. *JAMA Netw Open*. Published December 03, 2024. doi:10.1001/jamanetworkopen.2024.48601

Data
Data available: Yes
Data types: Deidentified participant data, Other (please specify)
Additional Information: Anonymized data will be shared by request from a qualified academic investigator for the sole purpose of replicating procedures and results presented in the article if data transfer is in agreement with relevant legislation on the general data protection regulation and decisions and by the relevant Ethical Review Boards, which should be regulated in a material transfer agreement.
How to access data: data will be shared under conditions stated above
When available: With publication

Supporting Documents
Document types: None

Additional Information
Who can access the data: Anonymized data will be shared by request from a qualified academic investigator for the sole purpose of replicating procedures and results presented in the article if data transfer is in agreement with relevant legislation on the general data protection regulation and decisions and by the relevant Ethical Review Boards, which should be regulated in a material transfer agreement.
Types of analyses: Raw MRI data, raw neuropsychological data
Mechanisms of data availability: will be agreed on with researchers requesting the data
Any additional restrictions: stated above
